# Supplementary material for: Is Structured Exercise Performed with Supplemental Oxygen a Promising Method of Personalized Medicine in the Therapy of Chronic Diseases?
Source: J Pers Med. 2020 Sep 19;10(3):135. doi: 10.3390/jpm10030135 (PMC7564446; doi:10.3390/jpm10030135)
Supplement: Supplementary file 1 [file jpm-10-00135-s001.pdf]

SUPPLEMENTS

Table 1 Overview of the search strategy

Search No.	Query	Hits
**PubMed/MedLine**		
#1	"Humans"[Mesh]	18,140,602
#2	"Exercise"[Mesh]	186,020
#3	"Exercise Tolerance"[Mesh]	12,154
#4	"Exercise Therapy"[Mesh]	48,194
#5	"Oxygen Inhalation Therapy"[Mesh]	25,350
#6	"Hyperoxia"[Mesh]	3,587
#7	"Oxygen/therapeutic use*"[Mesh]	12,652
#8	(("Exercise"[Mesh]) OR "Exercise Tolerance"[Mesh]) OR "Exercise Therapy"[Mesh]	223,795
#9	(("Oxygen Inhalation Therapy"[Mesh]) OR "Hyperoxia"[Mesh]) OR “Oxygen/therapeutic use*”[Mesh]	39,368
#10	(("Humans"[Mesh]) AND ((("Exercise"[Mesh]) OR "Exercise Tolerance"[Mesh]) OR "Exercise Therapy"[Mesh])) AND ((("Oxygen Inhalation Therapy"[Mesh]) OR "Hyperoxia"[Mesh]) OR “Oxygen/therapeutic use*”[Mesh])	**747**
**EMBASE**		
#1	'human'/exp	21,362,327
#2	'exercise'/exp	336,615
#3	'exercise tolerance'/exp	16,563
#4	'kinesiotherapy'/exp	77,848
#5	'oxygen therapy'/exp	49,095
#6	'hyperoxia'/exp	9,367
#7	#2 OR #3 OR #4	390,978
#8	#5 OR #6	57,496
#9	#1 AND #7 AND #8	**2,324**
**CINAHL**		
#1	(MH “Human”)	1,998,279
#2	(MH “Exercise”)	45,758
#3	(MH “Therapeutic Exercise”)	20,840
#4	(MH “Exercise Tolerance”)	4,233
#5	(MH “Oxygen Therapy”)	5,198
#6	(MH “Hyperoxia”)	489
#7	#2 OR #3 OR #4	68,867
#8	#5 OR #6	5,600
#9	#1 AND #7 AND #8	**65**
**ISI Web of Knowledge**		
#1	TS=humans	3,030,389
#2	TS=exercise	365,662
#3	TS=exercise therapy	39,925
#4	TS=exercise tolerance	13,416

#5	TS=oxygen inhalation therapy	1,083
#6	TS=oxygen therapy	48,726
#7	TS=hyperoxia	8,068
#8	#4 OR #3 OR #2	365,662
#9	#7 OR #6 OR #5	55,811
#10	#9 AND #8 AND #1	**468**
**Cochrane Library**		
#1	MeSH descriptor [Humans]	8,395
#2	MeSH descriptor [Exercise]	22,836
#3	MeSH descriptor [Exercise Tolerance]	2,470
#4	MeSH descriptor [Exercise Therapy]	12,598
#5	MeSH descriptor [Hyperbaric Oxygenation]	388
#6	MeSH descriptor [Oxygen Inhalation Therapy]	1,464
#7	MeSH descriptor [Hyperoxia]	180
#8	#2 OR #3 OR #4	30,807
#9	#5 OR #6 OR #7	1,621
#10	#1 AND #8 AND #9	**6**
**Grey Literature using Google Scholar**		
#1	Keyword search/hand search: exercise AND (hyperoxia OR oxygen therapy)	Stopped at **514**

Table 2 Overview of the outcomes of interest

Item	Rooyackers	Fichter	Garrod	Wadell	Emtner	Karlsen	Bjørgen	Scorsone	Ringbaek	Spielmanns	Neunhäuserer	Alison	Total
**W_{max}**	X	X			X	X	X	X		X	X		8
**VO_{2peak}**	X	X			X	X	X	X		X	X		8
**HR_{peak}**	X	X		X	X	X	X	X			X		8
**BLa**		X		X	X	X	X			X	X		7
**6MWT**	X			X[$]						X			3
**TTE**	X				X			X[#]					3
**ESWT**									X			X	2
**ISWT**			X						X[ß]			X	1
**Stair climb**	X												1
**Weight lifted**	X												1
**RPE**				X		X	X						3
**Dyspnea**	X		X[#]	X	X			X					5
**Leg Fatigue**					X			X					2
**CRDQ**	X		X[#]		X[#]							X	4
**HADS**			X								X		2
**LCADL**			X										1
**SF-36**					X[#]	X[#]				X[#]			3
**Macnew**						X							1
**SGRQ**									X				1
**Dyspnoea-12**												X	1
**TOTAL**	9	4	5	5	9	7	5	6	2	5	5	4	

6MWT six minute walking test; **BLa** blood lactate concentration; **CRDQ** Chronic Respiratory Disease Questionnaire; **Dyspnoea-12** Dyspnoea-12 questionnaire; **ESWT** endurance shuttle walk test; **HADS** Hospital Anxiety and Depression Scale; **HR_{peak}** peak heart rate; **ISWT** incremental shuttle walk test; **LCADL** London Chest Activity of Daily Living Scale; **Macnew** Heart Disease Health-related Quality of Life Questionnaire; **RPE** rating of perceived exertion; **SF-36** 36-Item Short Form Health Survey; **SGRQ** St. George's Respiratory Questionnaire; **TTE** time to exhaustion; **VO_{2peak}** peak oxygen consumption; **W_{max}** maximal watt. Note: [$] non-motorized treadmill; [ß] assessed only at baseline; [#] insufficient reporting and/or no response to provide missing values and therefore not considered in pooled analysis

Table 3 Summary of changes in physical fitness, functional capacity and patient-reported outcomes presented by included studies (chronological order of publication date)

Author (year)	Outcomes	Results: Within-group changes	Results: Between-group changes	Safety and Attendance [%]
Rooyackers et al. 1997	W_{max} [W]	SO: 58 ± 33 to 65 ± 39;<br>Normoxia: 70 ± 51 to 87 ± 58 (p<0.01)	SO ↔ Normoxia	Safety: no exercise- or supplemental oxygen-related dropouts reported<br><br>Attendance: Not reported
	VO_{2peak} [L · minute^{-1}]	SO: 1.0 ± 0.3 to 1.0 ± 0.3<br>Normoxia: 1.2 ± 0.5 to 1.2 ± 0.6	SO ↔ Normoxia	
	HR_{peak} [beats · minute^{-1}]	SO: 126 ± 17 to 126 ± 19<br>Normoxia: 133 ± 21 to 132 ± 23	SO ↔ Normoxia	
	6MWT [m]	SO: 389 ± 140 to 475 ± 180 (p<0.01)<br>Normoxia: 487 ± 191 to 610 ± 166 (p<0.01)	SO ↔ Normoxia	
	TTE [minutes]	SO: 4.5 ± 2.5 to 6.7 ± 3.7<br>Normoxia: 6.5 ± 4.4 to 6.5 ± 4.4	SO ↔ Normoxia	
	Dyspnoea (BORG 0-10)	SO: 6.6 ± 2.1 to 5.3 ± 1.2<br>Normoxia: 7.3 ± 2.4 to 5.8 ± 1.9	SO ↔ Normoxia	
	Stair climb [n]	SO: 22 ± 10 to 30 ± 14 (p<0.01)<br>Normoxia: 29 ± 12 to 41 ± 18 (p<0.01)	SO ↔ Normoxia	
	Weight lifted [n]	SO: 37 ± 9 to 46 ± 7 (p<0.01)<br>Normoxia: 36 ± 14 to 52 ± 18 (p<0.01)	SO ↔ Normoxia	
	CRDQ			
	Dyspnoea	SO: 15.9 ± 5.3 to 21.6 ± 5.5 (p<0.01)<br>Normoxia: 14.9 ± 5.8 to 21.6 ± 4.5 (p<0.01)	SO ↔ Normoxia	
	Fatigue	SO: 15.5 ± 4.3 to 18.8 ± 3.9<br>Normoxia: 17.4 ± 4.8 to 20 ± 4.5	SO ↔ Normoxia	
	Emotional function	SO: 29.5 ± 7.1 to 35.3 ± 6.3 (p<0.01)<br>Normoxia: 32 ± 6.9 to 35.2 ± 8.5	SO ↔ Normoxia	
	Mastery	SO: 18 ± 5.5 to 22.1 ± 3.3 (p<0.01)<br>Normoxia: 20.4 ± 4.1 to 22.8 ± 3.7 (p<0.01)	SO ↔ Normoxia	
	Total	SO: 79 ± 18 to 98 ± 16 (p<0.01)<br>Normoxia: 85 ± 16 to 100 ± 17 (p<0.01)	SO ↔ Normoxia	
Fichter et al. 1999	W_{max} [W]	SO: 65.4 ± 11.9 to 75.8 ± 21<br>Normoxia: 62.3 ± 23.8 to 86.3 ± 28.4 (p<0.05)	SO ↔ Normoxia	Safety: no exercise- or supplemental oxygen-related dropouts reported<br><br>Attendance: Not reported
	VO_{2peak} [L · minute^{-1}]	SO: 0.96 ± 0.14 to 1.12 ± 0.17<br>Normoxia: 1.11 ± 0.31 to 1.30 ± 0.45	SO ↔ Normoxia	
	HR_{peak} [beats · minute^{-1}]	SO: 126 ± 6 to 138 ± 10<br>Normoxia: 115 ± 11 to 120 ± 8	SO ↔ Normoxia	
	BLa [mmol · L^{-1}]	SO: 3.5 ± 0.9 to 3.6 ± 0.7<br>Normoxia: 3.98 ± 1.33 to 5.7 ± 1.1	SO ↔ Normoxia	
Garrod et al. 2000	SWT [m]	SO: PRE 160 ± 89.1, POST Δ +20<br>Normoxia: PRE 131 ± 103, POST Δ +43.63	SO ↔ Normoxia: -23.6 (95% CI: -70.7 to 23.5), p = 0.19	Safety: no exercise- or supplemental oxygen-related dropouts reported
	Dyspnoea (BORG 0-10)	SO: PRE 4.53 ± 1.12, POST Δ-1<br>Normoxia: PRE 3.83 ± 1.27, POST Δ +0.46	SO↓ vs. Normoxia: -1.46 (95% CI: -2.72 to -0.19), p = 0.02	
	CRDQ			
	Dyspnoea	SO: Δ +2.18; Normoxia: Δ +0.50	SO ↔ Normoxia: 1.68 (95% CI -1.04 to 4.4), p = 0.21	
	Fatigue	SO: Δ +1.27; Normoxia: Δ +0.37	SO ↔ Normoxia: 0.9 (95% CI -2.53 to 4.35), p = 0.58	
	Emotional function	SO: Δ +4.27; Normoxia: Δ +2.55	SO ↔ Normoxia: 1.73 (95% CI -3.18 to 6.64), p = 0.47	

	Mastery	SO: Δ +1.54; Normoxia: Δ +1.27	SO ↔ Normoxia: 0.27 (95% CI -2.11 to 2.65), p = 0.81	Attendance: Not reported
	Total	SO: PRE 84.4 ± 21.2, POST Δ +9.27<br>Normoxia: PRE 77.9 ± 24.4, POST Δ +5.6	SO ↔ Normoxia: 3.67 (95% CI -7.7 to 15.1), p = 0.50	
	HADS			
	Anxiety	SO: Δ -0.09; Normoxia: Δ -1.81	SO ↔ Normoxia: 1.72 (95% CI -0.59 to 4.05), p = 0.13	
	Depression	SO: Δ -1.36; Normoxia: Δ -1.36	SO ↔ Normoxia: 0.00 (95% CI -2.90 to 2.90), p = 0.49	
	Total	SO: PRE 13.5 ± 7.76, POST Δ -1.46<br>Normoxia: PRE 15.2 ± 9.9, POST Δ -4.64	SO ↔ Normoxia: 1.73 (95% CI -2.32 to 5.78), p = 0.38	
	LCADL			
	Self-care	SO: Δ -2.09; Normoxia: Δ -0.73	SO ↔ Normoxia: -1.36 (95% CI -3.46 to 0.73), p = 0.19	
	Domestic	SO: Δ -2.18; Normoxia: Δ -2.27	SO ↔ Normoxia: 0.09 (95% CI -3.89 to 4.06), p = 0.96	
	Physical	SO: Δ -1.00; Normoxia: Δ -0.27	SO ↔ Normoxia: -0.73 (95% CI -1.96 to 0.50), p = 0.23	
	Leisure	SO: Δ -1.54; Normoxia: Δ -1.36	SO ↔ Normoxia: -0.18 (95% CI -1.87 to 1.49), p = 0.82	
	Total	SO: PRE 36.3 ± 12.6, POST Δ -6.82<br>Normoxia: PRE 36.6 ± 14.4, POST Δ -4.64	SO ↔ Normoxia: -2.18 (95% CI -7.15 to 2.79), p = 0.37	
Wadell et al. 2001	HR_{peak} [beats · minute^{-1}]	SO: 128 (104 - 142) to 128 (102 - 140), %Δ 4.9<br>Normoxia: 137 (102 - 142) to 144 (113-155), %Δ 5.4	SO ↔ Normoxia	Safety: no exercise- or supplemental oxygen-related dropouts reported<br><br>Attendance: Not reported
	BLa [mmol · L^{-1}]	SO: 1.208 ± 0.339 to 2.388 ± 0.708 (p<0.05)<br>Normoxia: 1.204 ± 0.323 to 3.224 ± 1.799 (p<0.05)	SO ↔ Normoxia	
	6MWT [m]	SO: 210 (90 - 360) to 245 (140 - 380), %Δ 14.0 (p<0.01)<br>Normoxia: 230 (110 - 280) to 270 (130 - 390), %Δ 19.8 (p<0.01)	SO ↔ Normoxia	
	RPE (BORG 6-20)	SO: 15 (9 - 19) to 15 (10 - 17), %Δ 5.5<br>Normoxia: 16.5 (13 - 19) to 15 (12 - 17), %Δ -15.5 (p<0.05)	SO ↔ Normoxia	
	Dyspnea (BORG 0-10)	SO: 6.5 (3 - 10) to 4.5 (3-9), %Δ -5.0<br>Normoxia: 6.5 (4 - 9) to 6 (1 - 7), %Δ 0	SO ↔ Normoxia	
Emtner et al. 2003	W_{max} [W]	SO: 54 ± 25 to 67 ± 24 (p<0.05)<br>Normoxia: 54 ± 27 to 64 ± 29 (p<0.05)	SO ↔ Normoxia	Safety: no exercise- or supplemental oxygen-related dropouts reported<br><br>Attendance: Overall 99.7%
	VO_{2peak} [L · minute^{-1}]	SO: 0.89 ± 0.22 to 0.93 ± 0.27<br>Normoxia: 0.91 ± 0.36 to 0.97 ± 0.32	SO ↔ Normoxia	
	HR_{peak} [beats · minute^{-1}]	SO: 125 ± 22 to 128 ± 22<br>Normoxia: 125 ± 16 to 130 ± 19	SO ↔ Normoxia	
	BLa [mmol · L^{-1}]	SO: 4.2 ± 2.8 to 4.8 ± 2.3<br>Normoxia: 3.8 ± 1.8 to 4.6 ± 2.2	SO ↔ Normoxia	
	TTE [minutes]	SO: 6.6 ± 3.0 to 21.4 ± 10.1 (p<0.05)<br>Normoxia: 5.8 ± 2.4 to 16.7 ± 8.0 (p<0.05)	SO ↔ Normoxia	
	Dyspnoea (BORG 0-10)	SO: 6.3 ± 2.5 to 6.7 ± 2.1<br>Normoxia: 5.8 ± 1.8 to 5.9 ± 1.5	SO ↔ Normoxia	
	Leg fatigue (BORG 0-10)	SO: 5.4 ± 2.4 to 4.6 ± 2.7<br>Normoxia: 5.3 ± 2.2 to 4.0 ± 2.4	SO ↔ Normoxia	
	CRDQ			
	Dyspnoea	SO: Δ +7 (p<0.05); Normoxia: Δ +7 (p<0.05)	SO ↔ Normoxia	
	Fatigue	SO: Δ +5 (p<0.05); Normoxia: Δ +4 (p<0.05)	SO ↔ Normoxia	
	Emotional function	SO: Δ +7 (p<0.05); Normoxia: Δ +5 (p<0.05)	SO ↔ Normoxia	
	Mastery	SO: Δ +5 (p<0.05); Normoxia: Δ +4 (p<0.05)	SO↑ (p<0.05) vs. Normoxia	
	Total	SO: Δ +24 (p<0.05); Normoxia: Δ +20 (p<0.05)	SO ↔ Normoxia	
	SF-36			
	General health	SO: Δ +11 (p<0.05); Normoxia: not reported	SO↑ (p<0.05) vs. Normoxia	
	Vitality	SO: Δ +14 (p<0.05); Normoxia: Δ +13 (p<0.05)	SO ↔ Normoxia	
	Physical functioning	SO: Δ +7 (p<0.05); Normoxia: not reported	SO ↔ Normoxia	

	Role-Physical	SO: Δ +23 (p<0.05); Normoxia: not reported	SO ↔ Normoxia	
Karlsen et al. 2008	W_{max} [W]	SO: 123 ± 27 to 159 ± 37 (p<0.05)<br>Normoxia: 155 ± 38 to 204 ± 21 (p<0.05)	SO ↔ Normoxia	Safety: no exercise- or supplemental oxygen-related dropouts reported<br><br>Attendance: Supplemental oxygen: 29.6 ± 0.7 from 30 sessions<br>Normoxia: 29.1 ± 1.1 from 30 sessions
	VO_{2peak} [L · minute^{-1}]	SO: 2.11 ± 0.35 to 2.44 ± 0.48 (p<0.05)<br>Normoxia: 2.17 ± 0.53 to 2.53 ± 0.61 (p<0.05)	SO ↔ Normoxia	
	HR_{peak} [beats · minute^{-1}]	SO: 141 ± 19 to 152 ± 19 (p<0.05)<br>Normoxia: 163 ± 19 to 160 ± 19	SO ↔ Normoxia	
	BLa [mmol · L^{-1}]	SO: 3.61 ± 1.01 to 6.90 ± 1.57 (p<0.05)<br>Normoxia: 4.3 ± 1.2 to 6.23 ± 1.8	SO ↔ Normoxia	
	RPE (BORG 6-20)	SO: 18 ± 1 to 18 ± 1<br>Normoxia: 15 ± 2 to 17 ± 1 (p<0.05)	PRE SO↑ vs. PRE Normoxia (p<0.05),<br>SO vs. Normoxia↑ (p<0.05)	
	SF-36			
	General health	SO: not reported; Normoxia: not reported	Not reported	
	Vitality	SO: not reported; Normoxia: not reported	Not reported	
	Physical functioning	SO: not reported; Normoxia: not reported	Not reported	
	Role-Physical	SO: not reported; Normoxia: not reported	Not reported	
	Macnew			
	Physical	SO: %Δ +10 (p<0.05); Normoxia: %Δ +13 (p<0.05)	SO ↔ Normoxia	
	Emotional	SO: not reported; Normoxia: not reported	SO ↔ Normoxia	
	Social	SO: %Δ +9 (p<0.05); Normoxia: %Δ +10 (p<0.05)	SO ↔ Normoxia	
	Global	SO: not reported; Normoxia: %Δ +9 (p<0.05)	SO ↔ Normoxia	
Bjørgen et al. 2009	**One legged cycling**			Safety: no exercise- or supplemental oxygen-related dropouts reported<br><br>Attendance: Not reported
	W_{max} [W]	SO: 65 ± 40 to 85 ± 40<br>Normoxia: 55 ± 20 to 75 ± 30 (p<0.05)	SO ↔ Normoxia	
	VO_{2peak} [L · minute^{-1}]	SO: 1.3 ± 0.68 to 1.46 ± 0.58 (p<0.05)<br>Normoxia: 1.12 ± 0.43 to 1.32 ± 0.71 (p<0.05)	SO ↔ Normoxia	
	HR_{peak} [beats · minute^{-1}]	SO: 153 ± 72 to 159 ± 36<br>Normoxia: 142 ± 54 to 136 ± 81	SO ↔ Normoxia	
	BLa [mmol · L^{-1}]	SO: 5 ± 4.22 to 5.36 ± 4.63<br>Normoxia: 2.76 ± 1.62 to 3.56 ± 4.01	PRE SO↑ vs. PRE Normoxia (p<0.05)<br>POST SO↑ vs. POST Normoxia (p<0.05)	
	RPE (BORG 6-20)	SO: 16 ± 3 to 17 ± 5<br>Normoxia: 16 ± 4 to 17 ± 1	SO ↔ Normoxia	
	**Two legged cycling**			
	W_{max} [W]	SO: 100 ± 70 to 120 ± 70 (p<0.05)<br>Normoxia: 90 ± 40 to 110 ± 65 (p<0.05)	SO ↔ Normoxia	
	VO_{2peak} [L · minute^{-1}]	SO: 1.44 ± 0.62 to 1.68 ± 0.82 (p<0.05)<br>Normoxia: 1.42 ± 0.59 to 1.53 ± 0.74 (p<0.05)	SO ↔ Normoxia	
	HR_{peak} [beats · minute^{-1}]	SO: 162 ± 48 to 160 ± 36<br>Normoxia: 148 ± 69 to 142 ± 79	SO ↔ Normoxia	
	BLa [mmol · L^{-1}]	SO: 6.92 ± 5.25 to 4 ± 4.74<br>Normoxia: 2.44 ± 2.45 to 3.76 ± 2.52	SO vs. Normoxia↑ (p<0.05)	
	RPE (BORG 6-20)	SO: 17 ± 3 to 17 ± 5<br>Normoxia: 17 ± 1 to 19 ± 1	SO ↔ Normoxia	
Scorsone et al. 2010	W_{max} [W]	SO: 67 ± 21 to 76 ± 25 (p<0.01)<br>Normoxia: 82 ± 33 to 92 ± 35 (p<0.01)	SO ↔ Normoxia: p = 0.372	Safety: no exercise- or

	VO_{2peak} [L · minute^{-1}]	SO: 0.95 ± 0.31 to 1.05 ± 0.30	SO ↔ Normoxia: p = 0.159	supplemental
		Normoxia: 1.35 ± 0.60 to 1.41 ± 0.57		oxygen-related
	HR_{peak} [beats · minute^{-1}]	SO: 119 ± 25 to 118 ± 21	SO ↔ Normoxia: p = 0.850	dropouts
		Normoxia: 119 ± 19 to 120 ± 15		reported
	TTE [minutes]	SO: Δ +6.9 ± 4.4 (p<0.05)	SO ↔ Normoxia: p = 0.635	
		Normoxia: Δ +7.6 ± 4.9 (p<0.02)		Attendance: Not
	Dyspnoea (BORG 0-10)	SO: 7 ± 3 to 6 ± 3	SO ↔ Normoxia: p = 0.147	reported
		Normoxia: 7 ± 3 to 4 ± 2		
	Leg fatigue (BORG 0-10)	SO: 9 ± 1 to 7 ± 2	SO ↔ Normoxia: p = 0.799	
		Normoxia: 8 ± 2 to 7 ± 2		
Ringbaek et	**Week 0-7**			Safety: no
al. 2013	ESWT [m]	SO: 119.6 ± 75.7 to 168 ± 183 (p<0.01)	SO ↔ Normoxia: p = 0.77	exercise- or
		Normoxia: 148.5 ± 88.7 to 167 ± 239 (p<0.01)		supplemental
	SGRQ			oxygen-related
	Symptoms	SO: not reported; Normoxia: not reported		dropouts
	Activity	SO: not reported; Normoxia: not reported		reported
	Impacts	SO: not reported; Normoxia: not reported		
	Total	SO: PRE 62.1 ± 12.4, POST Δ -1.8 ± 8.0	SO ↔ Normoxia: p = 0.80	Attendance:
		Normoxia: 58.4 ± 11.9, POST Δ -3.2 ± 7.2		Overall: 88%;
	**Week 0-20**			Supplemental
	ESWT [m]	SO: 119.6 ± 75.7 to 170 ± 257 (p<0.01)	SO ↔ Normoxia: p = 0.83	oxygen: 89%;
		Normoxia: 148.5 ± 88.7 to 184 ± 314 (p<0.01)		Normoxia: 87%
	SGRQ week 0-20			In first 7 weeks
	Symptoms	SO: not reported; Normoxia: not reported		
	Activity	SO: not reported; Normoxia: not reported		
	Impacts	SO: not reported; Normoxia: not reported		
	Total	SO: PRE 62.1 ± 12.4, POST Δ -3.5 ± 7.2	SO ↔ Normoxia: p = 0.81	
		Normoxia: 58.4 ± 11.9, POST Δ -2.9 ± 8.6		
Spielmanns et	W_{max} [W]	SO: 52.6 ± 24.6 to 63 ± 26.9 (p<0.001) to 63.7 ± 33.7 (p<0.005)	SO ↔ Normoxia	Safety: no
al. 2015		Normoxia: 54.9 ± 22.4 to 68.4 ± 26.9 (p<0.001) to 68.7 ± 25.7 (p<0.004)		exercise- or
	VO_{2peak} [ml · kg^{-1} · minute^{-1}]	SO: 13.9 ± 3 to 15.3 ± 3.4 (p<0.005) to 15.5 ± 4.2 (p<0.08)	SO ↔ Normoxia	supplemental
		Normoxia: 13.9 ± 2.7 to 14.8 ± 3.5 (p<0.15) to 14.7 ± 4.1 (p<0.71)		oxygen-related
	BLa [mmol · L^{-1}]	SO: 3.3 ± 0.93 to 2.9 ± 0.93 to 3.0 ± 0.89	SO ↔ Normoxia	dropouts
		Normoxia: 3.2 ± 0.72 to 3.1 ± 0.74 to 2.7 ± 0.82		reported
	6MWT [m]	SO: 574 ± 98 to 630 ± 88 (p<0.002) to 647 ± 99 (p<0.001)	SO ↔ Normoxia	
		Normoxia: 551 ± 106 to 621 ± 76 (p<0.001) to 635 ± 63		Attendance: Not
	SF-36			reported
	General Health	only graphical; exact values not reported	SO ↔ Normoxia	however it was
	Vitality	only graphical; exact values not reported	SO ↔ Normoxia	stated that
	Physical functioning	only graphical; exact values not reported	SO ↔ Normoxia	participation had
	Role-Physical	only graphical; exact values not reported	SO ↔ Normoxia	to be 80%
	Mental Health	only graphical; exact values not reported	SO ↔ Normoxia	
	Role-Emotional	only graphical; exact values not reported	SO ↔ Normoxia	
	Bodily pain	only graphical; exact values not reported	SO ↔ Normoxia	
	Social functioning	only graphical; exact values not reported	SO ↔ Normoxia	
Neunhäuserer	W_{max} [W]	SO: 79.3 ± 30.8 to 94.0 ± 32.3 (p<0.01)	SO ↑ (0.024) vs. Normoxia	Safety: no
et al. 2016		Normoxia: 91.7 ± 31.8 to 100.5 ± 33.1 (p<0.01)		exercise- or
	VO_{2peak} [ml · kg^{-1} · minute^{-1}]	SO: 18.6 ± 3.6 to 20.9 ± 3.5 (p<0.05)	SO ↔ Normoxia	supplemental
		Normoxia: 18.3 ± 4.0 to 19.8 ± 4.4 (p<0.05)		oxygen-related

	HR_{peak} [beats · minute^{-1}]	SO: 132.2 ± 19.4 to 140.4 ± 18.2<br>Normoxia: 124.3 ± 15.7 to 128.7 ± 15.9	SO ↔ Normoxia	dropouts reported
	BLa [mmol · L^{-1}]	SO: 3.8 ± 1.2 to 5.3 ± 1.9<br>Normoxia: 3.8 ± 1.4 to 4.6 ± 1.9	SO ↔ Normoxia	Attendance: Not reported
	10-RM [kg]	No data provided before crossover	No data provided before crossover	
	HADS			
	Anxiety	No data provided before crossover	No data provided before crossover	
	Depression	No data provided before crossover	No data provided before crossover	
Alison et al. 2019	ESWT [sec]	SO: 327 ± 191 to 500 ± 361 ($p<0.05$)<br>Normoxia: 319 ± 139 to 456 ± 308 ($p<0.05$)	SO ↔ Normoxia: 15 (95% CI: -106 to 136)	Safety: Supplemental oxygen: n = 1 atrial fibrillation during training Normoxia: n = 1 mild stroke after finishing treadmill training session
	ISWT [m]	SO: 287 ± 121 to 326 ± 128 ($p<0.05$)<br>Normoxia: 285 ± 124 to 304 ± 132 ($p<0.05$)	SO ↔ Normoxia: 5 (95% CI: -14 to 25)	
	CRDQ			
	Dyspnoea	SO: 3.2 ± 1.0 to 3.8 ± 1.2 ($p<0.05$)<br>Normoxia: 2.9 ± 1.0 to 3.5 ± 1.1 ($p<0.05$)	SO ↔ Normoxia: 0.1 (95% CI: -0.3 to 0.5)	
	Fatigue	SO: 4.0 ± 1.1 to 4.5 ± 1.1 ($p<0.05$)<br>Normoxia: 3.5 ± 1.3 to 4.2 ± 1.3 ($p<0.05$)	SO ↔ Normoxia: 0.03 (95% CI: -0.4 to 0.5)	
	Emotional function	SO: 4.8 ± 1.1 to 5.2 ± 1.1 ($p<0.05$)<br>Normoxia: 4.8 ± 1.2 to 5.0 ± 1.2	SO ↔ Normoxia: 0.2 (95% CI: -0.2 to 0.5)	
	Mastery	SO: 5.0 ± 1.2 to 5.3 ± 1.2 ($p<0.05$)<br>Normoxia: 5.0 ± 1.4 to 5.4 ± 1.2 ($p<0.05$)	SO ↔ Normoxia: -0.1 (95% CI: -0.5 to 0.3)	Attendance: Not reported but patients had to complete 16 sessions (66%) to be included for analysis Supplemental oxygen: n = 48 Normoxia: n = 41
	Total	SO: 4.3 ± 0.8 to 4.7 ± 0.9 ($p<0.05$)<br>Normoxia: 4.1 ± 1.0 to 4.6 ± 0.9 ($p<0.05$)	SO ↔ Normoxia: 0.0 (95% CI: -0.3 to 0.3)	
	Dyspnoea-12			
	Physical	SO: 11 ± 6 to 9 ± 5 ($p<0.05$)<br>Normoxia: 12 ± 6 to 11 ± 6	SO ↔ Normoxia: -1.2 (95% CI: -2.9 to 0.5)	
	Affective	SO: 5 ± 4 to 4 ± 4<br>Normoxia: 5 ± 5 to 5 ± 5	SO ↔ Normoxia: -0.9 (95% CI: -2.2 to 0.4)	
	Total	SO: 15 ± 9 to 13 ± 9 ($p<0.05$)<br>Normoxia: 17 ± 9 to 17 ± 9	SO ↔ Normoxia: -1.9 (95% CI: -4.5 to 0.7)	

6MWT 6-minute walking test; *10-RM* ten repetition maximum; *BLa* blood lactate concentration; *CRDQ* Chronic Respiratory Disease Questionnaire; *Dyspnoea-12* Dyspnoea-12 questionnaire; *ESWT* endurance shuttle walk test; *HADS* Hospital Anxiety and Depression Scale; HR_{peak} peak heart rate; *ISWT* incremental shuttle walk test; *LCADL* London Chest Activity of Daily Living Scale; *Macnew* Heart Disease Health-related Quality of Life Questionnaire; *RPE* rating of perceived exertion; *SF-36* 36-Item Short Form Health Survey; *SGRQ* St. George's Respiratory Questionnaire; *SWT* shuttle walk test; *SO* supplemental oxygen; *TTE* time to exhaustion; VO_{2peak} peak oxygen consumption; W_{max} maximal watt
